# Successful Treatment for Adult Precursor B-Cell Lymphoblastic Lymphoma Involving Central Nervous System Monitored by ^18^F-Fluorodeoxyglucose PET/CT Imaging

**DOI:** 10.3389/fonc.2020.00334

**Published:** 2020-03-24

**Authors:** Xiang Shi, Weiyan Zhou, Mengqiao Xu, Tao Hua, Yihui Guan

**Affiliations:** ^1^Department of Ophthalmology, School of Medicine, Shanghai General Hospital, Shanghai JiaoTong University, Shanghai, China; ^2^PET Center, Huashan Hospital, Fudan University, Shanghai, China; ^3^Institute of Integrative Medicine, Fudan University, Shanghai, China

**Keywords:** precursor B-cell lymphoblastic lymphoma, fluorodeoxyglucose (FDG), PET/CT, Philadelphia chromosome, imatinib

## Abstract

Precursor B-cell lymphoblastic lymphoma (PBLL) is a rare subtype of non-Hodgkin lymphoma originating from B-cell precursors. PBLL, as a solitary mass lesion affecting the central nervous system without leukemic disease at presentation, is quite uncommon. Here we report a rare PBLL case with Philadelphia chromosome positivity. The 44-year-old male presented a solitary bulky mass primarily involving the left frontotemporal lobes and extended into the infratemporal fossa. Pretreatment PET/CT imaging showed avid ^18^F-fluorodeoxyglucose (^18^F-FDG) uptake of the lesion. By aggressive chemotherapy and imatinib maintenance treatment, the patient achieved and remained in complete remission on another two consecutive PET/CT imaging follow-ups.

## Case Presentation

A previously healthy 44-year-old male presented with chronic vision loss of the left eye for 1 month and complained about progressive distending pain of his left orbital region. Ophthalmic examinations found that his left eye was exophthalmic with positive relative afferent pupillary defect (RAPD), which indicated optic nerve injury. The best-corrected visual acuity (BCVA) of his left eye was 0.4. Unenhanced orbital CT detected an intracranial mass lesion with concurrent extracranial invasion. Malignancy was highly suspected. The patient underwent brain MRI and whole-body 18F-fluorodeoxyglucose (^18^F-FDG) PET/CT.

**Figure 1:**
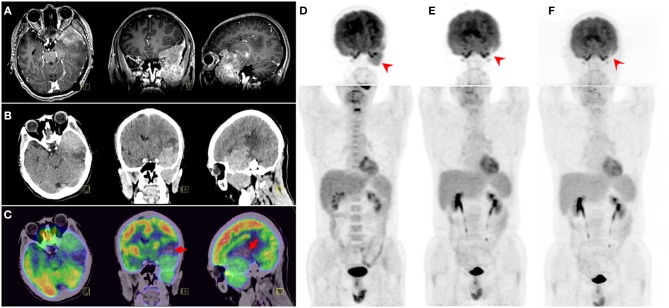
MRI revealed a heterogeneous enhanced mass lesion with meningeal and adjacent frontal-temporal lobes involvement **(A)**. CT displayed a heterogeneous hyper-intensity mass lesion and peripheral edema **(B)**. 18F-fluorodeoxyglucose (^18^F-FDG) PET/CT showed intense FDG uptake with SUV_max_ of 12.0 in the left frontal–temporal lobes. Part of the lesion was naive with FDG uptake (arrows) **(C)**, which could be caused by possible intratumoral hemorrhage. Adjacent skull base bone destruction and extracranial extension to the left infratemporal fossa were also observed. Maximum intensity projection (MIP) view of pretreatment ^18^F-FDG PET/CT **(D)** showed the solitary bulky mass. Two consecutive ^18^F-FDG PET/CT follow-ups **(E,F)** demonstrated complete remission.

MRI revealed a heterogeneous enhanced mass lesion with meningeal and adjacent frontal–temporal lobes involvement (Figure 1A). The mass lesion showed heterogeneous hyperdensity on CT images, and peripheral edema was observed (Figure 1B). ^18^F-FDG PET/CT showed intense FDG uptake with maximum standardized uptake value (SUV_max_) of 12.0 in the left frontal–temporal lobes (Figure 1C). No evident bone marrow (BM) involvement was observed on ^18^F-FDG PET/CT (Figure 1D). Lymphadenopathy and hepatosplenomegaly were also absent.

**Figure 2:**
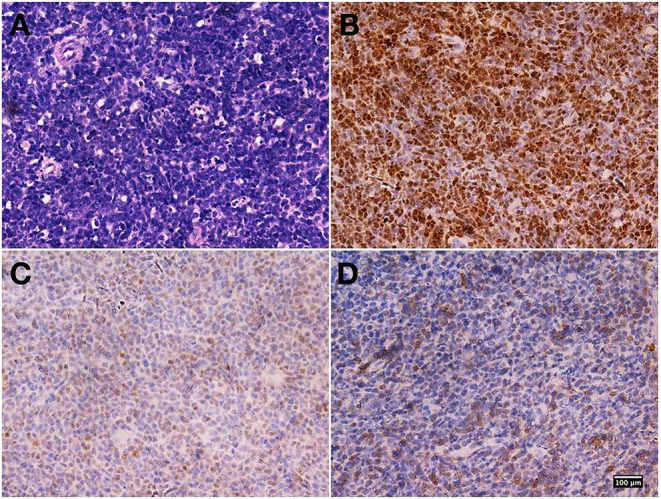
Biopsy specimen pathological findings. Hematoxylin and eosin staining revealed diffusely distributed atypical lymphoid cells **(A)**. Immunohistochemical staining showed that the oval-round tumor cells were positive for paired box 5 (PAX5) **(B)**, terminal deoxynucleotidyl transferase (TdT) **(C)**, and CD79a **(D)**. Magnification, ×400 for all.

A biopsy tissue specimen of about 5 × 15 mm was obtained under MRI guidance for pathological diagnosis. Immunohistochemical staining showed that the oval-round tumor cells were positive for paired box 5 (PAX5), terminal deoxynucleotidyl transferase (TdT), and CD79a, which indicated the diagnosis of precursor B-cell lymphoblastic lymphoma (PBLL) (Figure 2). Ki-67 staining showed that the proportion of positive tumor cells was ~70%. BM aspiration and peripheral blood cell counts excluded acute lymphoblastic leukemia (ALL). Further karyotyping and analysis of breakpoint cluster region (BCR)/Abelson murine leukemia (ABL)1 genes (22q11/9q34) for biopsy specimen were positive at a level of 71%, indicating a possibly promising treatment of tyrosine kinase inhibitors (TKIs).

After the diagnosis of PBLL was confirmed, the patient received six-cycle intravenous chemotherapy with hyper-fractionated cyclophosphamide, vincristine, Adriamycin, and dexamethasone (hyper-CVAD) plus rituximab regimen. Additional intrathecal chemotherapy (methotrexate 10 mg, cytosine arabinoside 50 mg, dexamethasone 5 mg) for eight cycles was applied. A follow-up ^18^F-FDG PET/CT scan demonstrated a remarkable metabolism decline of the lesion after the treatment (Figure 1E). After remission, the patient received maintenance therapy with imatinib. A third ^18^F-FDG PET/CT scan 2-years after initial diagnosis also showed no relapse (Figure 1F). No abnormal signs were found on peripheral blood and BM aspiration smears, BM flow cytometry, cerebrospinal fluid (CSF) analyses, ultrasonography, MRI, and skeletal scintigraphy examinations.

## Discussion and Conclusions

PBLL is a rare subtype of non-Hodgkin lymphoma originating from B-cell precursors, as the name suggests. PBLL patients could have nodal or extranodal involvement, and BM involvement is <25% (1, 2). Precursor lymphoblastic lymphoma (PLL) can be of B-cell or T-cell lineage, and the clinical manifestation varies significantly. Precursor T-cell lymphoblastic lymphoma (PTLL) is more common in children, mainly presented with an anterior mediastinal mass. PBLL only accounts for 10–25% of all PLL and typically presents with subcutaneous, soft tissue, bone, and lymph node disease in children or young adults. Primary uncommon lesions of PBLL in testicles, localized skin, pancreas, and musculoskeletal system on ^18^F-FDG PET imaging were reported, with SUV_max_ ranging from 3.7 to 19.1 (3–9). Besides that, there are also case reports with initial symptoms of ovaries, retroperitoneum, tonsil, uterus, stomach, colon, mediastinum, and both lytic and blastic bone lesions (10–13).

PBLL presenting as one solitary mass lesion affecting the central nervous system (CNS) without leukemic disease is quite rare but could be more aggressive with a worse prognosis. Despite prophylactic treatment, some PBLL patients still relapse in the CNS. Diffuse leptomeningeal infiltration after the confirmation of diagnosis or relapse is also common. Presentation of leptomeningeal lymphoblastic lymphoma newly diagnosed in a 6-year-old boy was reported (14). However, a mass lesion affecting the CNS without leukemic disease as an initial manifestation of PBLL is rare. To our knowledge, there is only one case report of a PBLL patient who presented with compression of spinal cord mass lesion (15). Differential diagnoses, including malignant meningioma, sarcomas of muscle or bone origin, and metastatic tumors, should be considered for this patient.

In this case, we were able to exclude myeloid, T-cell, and natural killer (NK)/T-cell lineage because the malignant cells did not express the myeloid marker myeloperoxidase, the T-cell marker CD3, or the NK/T-cell marker CD56. By contrast, this patient's immunohistochemistry stain showed positive B-cell markers, including PAX5, TdT, and CD79a, but absent for CD20, which indicated the origin of B-cell lineage. Philadelphia (Ph) chromosomes present in 90–95% of chronic lymphocytic leukemia (CML) patients, but less frequently in PBLL/ALL patients. Ph-positive PBLL is rare (16). Based on the patient's cell/molecular genetic changes and the treatment of Ph-chromosomal-positive ALL, imatinib was added at the maintaining time of chemotherapy to obtain better efficacy. The patient remained in complete remission at the last follow-up.

This case exemplifies the importance of cytogenetic or molecular genetic analysis for the diagnosis and treatment of PBLL/ALL. As a molecular and functional imaging method, ^18^F-FDG PET/CT could facilitate lesion detection and hematologic tumor evaluation after aggressive chemotherapy and TKIs maintaining intervention.

## Data Availability Statement

All datasets generated for this study are included in the article/supplementary material.

## Ethics Statement

The study was approved by the Human Ethics Review Committee of Huashan Hospital, Fudan University. A written informed consent to publish the report and associated medical images was obtained from the patient.

## Author Contributions

All authors listed have made a substantial, direct and intellectual contribution to the work, and approved it for publication.

### Conflict of Interest

The authors declare that the research was conducted in the absence of any commercial or financial relationships that could be construed as a potential conflict of interest.
